# Dietary Theabrownins Improve Freeze–Thaw Quality of Grass Carp (*Ctenopharyngodon idella*) Muscle by Inhibiting Ice Crystal Growth and Modulating Metabolism

**DOI:** 10.3390/foods15030462

**Published:** 2026-01-28

**Authors:** Wei Zeng, Xuexue Zhang, Bohua Wang, Song Lei, Huan Zhong, Yi Zhou, Zehong Wei

**Affiliations:** 1 College of Life Sciences, Hunan Normal University, Changsha 410081, China; zengwei_2014@163.com (W.Z.); zxx18251611150@126.com (X.Z.); zhonghuanzh@126.com (H.Z.); 2 College of Life and Environmental Sciences, Hunan University of Arts and Science, Changde 415000, China; wangbohua0731@126.com (B.W.); leidasong@163.com (S.L.)

**Keywords:** theabrownins, *Ctenopharyngodon idella*, freeze-thaw, metabolomics, muscle

## Abstract

Freezing is the most commonly used preservation method for aquatic products, but the freeze–thaw cycle leads to the formation and growth of ice crystals, which seriously affects the quality of fish. This study evaluated the effect of dietary supplementation with theabrownins (TBs) on the quality attributes of grass carp (*Ctenopharyngodon idella*) muscle following freeze–thaw treatment. We examined changes in textural properties, ice crystal morphology and metabolomic profiles in response to TBs inclusion in feed. The results indicated that feeding TBs at 0.02% and 0.06% levels significantly improved the chewiness and cohesiveness of grass carp muscle. Histological analysis revealed that TB-containing feed effectively inhibited ice crystal growth, leading to smaller and more uniform ice crystals, thereby mitigating structural damage to muscle tissue. Metabolomic analysis identified distinct metabolite profile differences between the treatment groups and the control group, with both LTB (0.02% TBs) and HTB (0.06% TBs) groups showing significant upregulation of esters and aromatic compounds compared to the control group. The present study demonstrates that TBs, as a natural feed additive, can enhance the freeze–thaw tolerance of grass carp muscle by modulating ice crystal formation and influencing muscle metabolism. This study provides important insights and practical implications for developing novel strategies to improve the quality of frozen aquatic products.

## 1. Introduction

The aquaculture industry is currently undergoing a remarkable development, driven by continuous innovations in production technologies, the increasingly pervasive adoption of digital management systems and a heightened emphasis on sustainability and environmental stewardship [1,2,3]. As a critical component of global food security, this industry plays an essential role in addressing the escalating worldwide demand for protein resources [4,5]. The current development of aquaculture has also promoted the advancement of various processing, preservation and analysis technologies [6]. The ongoing digital transformation characteristic of the Industry 4.0 era is further accelerating the innovation of these technologies [7]. Nevertheless, numerous critical issues remain to be addressed in the preservation and transportation of aquatic products.

Preservation by freezing is a widely adopted and critical method for maintaining the quality of aquatic products [8]. This technique effectively suppresses microbial growth and enzymatic activity, thereby significantly prolonging shelf-life (6–12 months), preserving nutritional quality and enabling long-distance transport and extended storage [8,9]. However, the freezing process itself can adversely affect the physicochemical and sensory attributes of aquatic products [10]. These detrimental effects commonly include moisture loss, textural deterioration, flavor and odor alterations and structural damage from ice crystal formation [8,10,11,12]. To mitigate these adverse effects, several ways have been developed, primarily focusing on optimizing the freezing process itself and employing auxiliary methods [13]. Current approaches include the application of rapid freezing techniques, vacuum packaging prior to freezing and ice glazing, all aimed at minimizing intracellular ice crystal formation and oxidative reactions [9,14,15]. Although the aforementioned techniques are effective in mitigating the deterioration of fish quality following freezing, their practical application is frequently limited by factors such as operational complexity, high costs and inconsistent efficacy across various fish species and sources [16,17].

Theabrownins (TBs) are a class of complex polymeric polyphenols generated primarily via the enzymatic or non-enzymatic oxidation of tea polyphenols during fermentation [18,19]. As one of the most abundant pigments in fermented teas, TBs are key contributors to the distinctive organoleptic properties (color and flavor) of these beverages [20,21]. TBs have been demonstrated to possess a spectrum of beneficial biological activities, including antioxidant, anti-inflammatory, metabolic syndrome-alleviating and neuroprotective effects [22]. Studies have shown that TBs attenuated hyperlipidemia by inhibited enzymatic activity of pancreatic lipase and cholesterol esterase, influenced micelle formation and decreased micellar cholesterol solubility [23]. TBs alleviate skeletal muscle inflammation by modulating the TLR4/MyD88/NF-κB signaling pathway and suppressing the expression of pro-inflammatory cytokines, indicating its potential therapeutic value in the prevention and treatment of chronic inflammatory diseases [24]. In addition, because the main component of TBs contains hydroxyl aromatic structures, TBs can form hydrogen bond networks with water molecules, thereby modifying the microenvironment of the local aqueous phase [18]. This interaction effectively delays ice nucleation and suppresses the subsequent growth of ice crystals. Similar cryoprotective effects have been observed in other natural polyphenols as well as sugar alcohols [25,26]. Furthermore, molecular simulation studies have demonstrated that certain biological macromolecules with defined spatial conformations can selectively adsorb onto specific crystallographic planes of ice, thereby impeding further crystal growth [27]. However, the systemic bioavailability of TBs remains relatively low, and its precise mechanism of action as well as its practical applications in the food industry warrant further in-depth investigation.

Grass carp (*Ctenopharyngodon idella*) is a widely distributed and economically significant fish species [28]. With the expansion of market demand, the output of grass carp farming has been increasing year by year. It occupies a prominent position in the global freshwater aquaculture industry [29,30]. However, grass carp is particularly susceptible to quality deterioration during frozen storage due to its high polyunsaturated fatty acid content and relatively high moisture content, which accelerates lipid oxidation and protein denaturation [31]. Moreover, the large-scale production and long-distance distribution of grass carp necessitate extended frozen storage periods, making it an ideal model for studying the effects of freeze–thaw on fish quality [32]. It is important to note that preservation by freezing exerts an irreversible effect on the quality of fish, making the mitigation of post-thaw quality deterioration an urgent challenge. Based on the documented bioactivities of polyphenolic compounds, we hypothesized that dietary TBs supplementation could improve the quality of grass carp under freeze–thaw treatment via regulating ice crystal morphology and muscle metabolism. To address this issue, the present study supplemented the diet of grass carp with varying concentrations of TBs. The effect of adding TBs to the feed was subsequently evaluated following freeze–thaw cycles through comprehensive assessments of quality characteristics, ice crystal morphology and metabolomic profiles.

## 2. Materials and Methods

### 2.1. TBs and Experimental Diets

To meet the nutritional requirements of grass carp, feed is formulated according to a specific ratio (Table 1). The TBs (purity > 98%) were obtained from the National Engineering Research Center for Botanical Functional Ingredients Utilization at Hunan Agricultural University. Prepared from fully fermented tea via enzymatic oxidative polymerization (20–55 °C, 2–4 h, pH 5.0–6.5), TBs were purified by ultrafiltration and adsorption, then concentrated and spray-dried to obtain TBs. During feed preparation, raw materials were ultra-finely ground and passed through an 80-mesh sieve to ensure uniform particle size. The homogenized mixture was then combined with water and extruded into buoyant pellets with a diameter of 1 mm using a single-screw extruder (Model EF-003, Zhuhai, China). All experimental diets were dehydrated in a forced-air drying oven at 65 °C, then stored at −20 °C to preserve nutritional integrity until use in the feeding trial.

### 2.2. Fish Rearing and Sampling

All the experimental fishes used in this experiment were taken from the Hunan Fish Genetics and Breeding Center of Hunan Normal University (Changsha, China). A total of 270 healthy grass carp with uniform size and an initial body weight of 23.5 ± 1.0 g were selected and randomly assigned to three experimental groups: the normal control group (NC), the 0.02% TBs group (LTB) and the 0.06% TBs group (HTB), with 90 fish in each group. Fish were fed twice daily at 08:00 and 16:00, with a feeding rate of 3% to 5% of their body weight, adjusted according to observed feeding behavior. During the breeding period, continuous aeration was maintained to ensure optimal water conditions, including a temperature of 25 ± 2 °C, dissolved oxygen levels exceeding 5.0 mg/L and ammonia nitrogen concentrations below 0.05 mg/L. The fishes were fed continuously for 8 weeks following the natural light cycle. They were fasted for 24 h and anesthetized with MS-222. Twelve fish were randomly selected from each group. After the skin was removed with a scalpel and forceps, the back muscle part was immediately taken and packed in a polyethylene bag and stored at −80 °C. At the outset of the freeze–thaw experiment, all samples were subjected to a complete freeze–thaw cycle under a standardized protocol. This cycle consisted of freezing at −20 °C for 12 h, followed by thawing at 4 °C for an additional 12 h.

### 2.3. Texture Analyzer

The dorsal muscle tissues of grass carp were excised following freezing and thawing, then cut into regular rectangular cubes measuring 1.0 cm × 1.0 cm × 0.5 cm. Each experimental group consisted of six replicates. After undergoing one freeze–thaw cycle, all texture samples were equilibrated at room temperature (20–25 °C) for a minimum of 30 min. The analysis was performed using a Texture Analyzer (FTC, TMS-Pilot, Sterling, VA, USA). Texture analysis was conducted under the following parameters: force transducer range of 250 N, trigger force of 0.1 N, test speed of 30 mm/min, deformation of 60% and recovery height of 10 mm. The mean values and standard deviations were calculated from all replicate measurements within each group.

### 2.4. Tissue Section Analysis

Hematoxylin-eosin (HE) sections were dehydrated through a graded ethanol series, cleared in xylene and embedded in paraffin. Serial sections with a thickness of 6 μm were cut using a rotary microtome (Leica RM2235, Baden-Württemberg, Germany). The sections were subsequently dewaxed in xylene and rehydrated through a descending ethanol series. Hematoxylin staining was performed for 5 min, followed by eosin staining for 1 min (Solarbio, Beijing, China). After dehydration through graded alcohols, the sections were mounted with neutral balsam (Beyotime, Shanghai, China). Bright-field images of HE sections were acquired using a Nikon Eclipse Ni microscope (Nikon, Tokyo, Japan). The ice crystal diameters were analyzed using Image J software(version 1.46 r), referring to previous studies for the analysis process [33].

### 2.5. CG-MS Analysis

A total of 50 mg of the sample was added to 100 μL of 50% methanol–water solution and ultrasonic extraction of metabolites was performed for 15 min. Subsequently, 100 μL of pure acetonitrile was added and mixed thoroughly. The mixture was allowed to stand at −20 °C for 1 h. The sample was centrifuged at 13,000 rpm for 15 min and the supernatant was collected for instrumental analysis. Metabolite detection was performed using an Agilent 8890 GC-5977MSD (Agilent Technologies, Santa Clara, CA, USA) gas chromatography–tandem mass spectrometry (GC-MS) system equipped with an automatic liquid sampler (ALS). The injection volume was 1 μL, and the splitless injection mode was employed with helium as the carrier gas at the inlet. The separation was achieved on a capillary column (P/N: 19091J-413; HP-5; 30 m × 320 μm × 0.25 μm) using high-purity nitrogen (99.999%) as the carrier gas in constant flow mode at a flow rate of 1 mL/min. The temperature program was set as follows: initial hold at 60 °C for 0.5 min, followed by a ramp of 15 °C/min to a final temperature of 330 °C, which was held for 3 min. The column was then cooled to 60 °C, resulting in a total chromatographic run time of 25 min. Electron ionization (EI) in positive mode (EI+) was used with an electron energy of 70 eV. The ion source temperature was maintained at 230 °C, the quadrupole temperature at 150 °C and the transfer line temperature at 280 °C. The electron multiplier detector operated in Gain Factor mode with a gain setting of 15. Mass spectral data were acquired in Profile mode over a scan range of 50–450 *m*/*z*. The GC-MS data acquired from the instrument were analyzed for qualitative and quantitative compound analysis using the Agilent MassHunter Qualitative Analysis Workflows (version 12.0) software. The resulting compound identifications and corresponding abundance values were subsequently used for downstream data analysis.

### 2.6. Statistical Analysis

All experimental data in this study were analyzed using SPSS Statistics version 22.0 software, with quantitative results presented as mean ± SD. *p* < 0.05 was considered statistically significant. In metabolomics analysis, principal component analysis (PCA) and partial least squares discriminant analysis (PLS-DA) were initially performed. Multidimensional data mining was conducted using PLS-DA and OPLS-DA. During data preprocessing, raw data were normalized using Pareto scaling, and a cross-validation procedure was applied to assess model reliability. In the construction of the OPLS-DA model, the variable importance in projection (VIP) values for each metabolite were calculated. By integrating the results of Student’s *t*-test and fold-change analysis, metabolites meeting both criteria VIP > 1 and *p* < 0.05 were identified as significantly differentially expressed.

## 3. Results

### 3.1. Supplementing TBs into the Feed of Farmed Grass Carp Enhances the Textural Quality of the Fish After Freeze–Thaw Cycles

**Figure 1:**
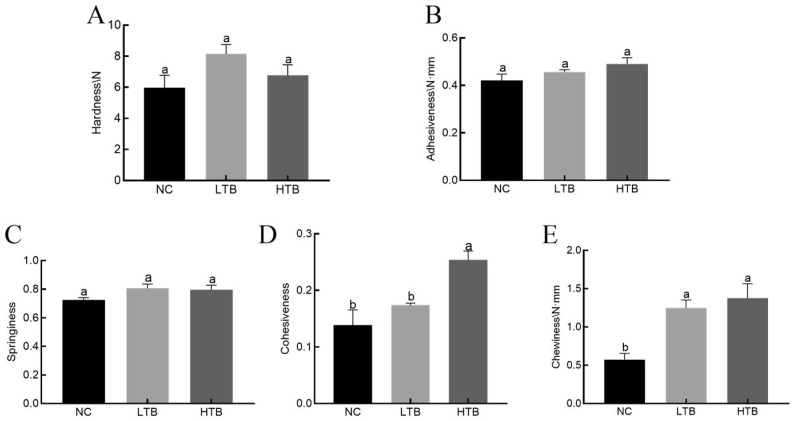
The effect of dietary supplementation with TBs on the texture characteristics of grass carp muscle following freeze–thaw cycles. (**A**) The effect of dietary supplementation with TBs on the hardness of grass carp muscle following freeze–thaw cycles. (**B**) The effect of dietary supplementation with TBs on the adhesiveness of grass carp muscle following freeze–thaw cycles. (**C**) The effect of dietary supplementation with TBs on the springiness of grass carp muscle following freeze–thaw cycles. (**D**) The effect of dietary supplementation with TBs on the cohesiveness of grass carp muscle following freeze–thaw cycles. (**E**) The effect of dietary supplementation with TBs on the chewiness of grass carp muscle following freeze–thaw cycles.The same letters indicate no significant difference between groups (*p* > 0.05), while different letters indicate a significant difference between groups (*p* < 0.05).

The results demonstrated that there were no significant differences in adhesiveness, hardness and springiness between the NC, LTB and HTB groups of fish muscle (Figure 1A–C). With regard to chewiness, both the 0.02% and 0.06% TBs groups exhibited improved values after freeze–thaw cycles (Figure 1D). In terms of cohesiveness, the HTB group showed a significant increase compared to both the NC and LTB groups (Figure 1E). Overall, supplementing 0.02% and 0.06% TBs in the feed did not affect the adhesion or hardness of grass carp following freeze–thaw cycles. Grass carp fed diets supplemented with TBs maintained favorable textural properties during frozen storage.

### 3.2. The Effects of TBs on the Morphological Characteristics of Ice Crystals in Grass Carp Muscle

**Figure 2:**
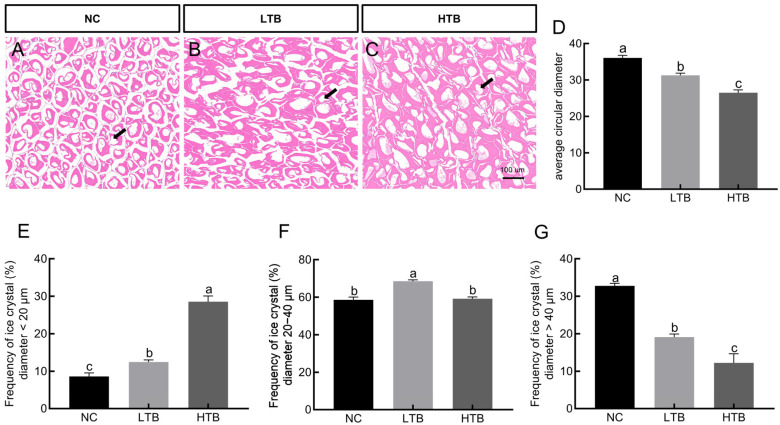
Histological analysis of grass carp fed with different concentrations of TBs. (**A**) HE optical micrographs of the NC group. (**B**) HE optical micrographs of the LTB group. (**C**) HE optical micrographs of the HTB group. (**D**) Analysis of the average diameter of ice crystals. (**E**) Transverse size distribution of ice crystals in the NC group. (**F**) Transverse size distribution of ice crystals in the LTB group. (**G**) Transverse size distribution of ice crystals in the HTB group. The black arrow indicates the position of the ice crystal. Different letters indicate statistically significant differences (*p* < 0.05).

The HE results indicated that the cellular architectures in both the LTB and HTB groups were better preserved, whereas the NC group (average circular diameter 36.05 μm) exhibited large ice crystals (Figure 2A–C). In contrast, both the LTB (average circular diameter 31.27 μm) and HTB groups (average circular diameter 26.52 μm) led to a significant refinement of ice crystal morphology, characterized by smaller size (Figure 2D). Furthermore, compared with the NC, the addition of TBs markedly reduced ice crystal diameter, with the high-concentration TBs demonstrating a more pronounced effect. Analysis of ice crystal size distribution revealed a greater proportion of crystals with diameters <20 μm in the HTB group, while the ice crystals in the NC group were within the range of >40 μm (Figure 2E–G). The results suggested that TBs effectively alleviated freeze–thaw-induced structural damage in grass carp muscle tissue.

### 3.3. Overall Characteristic Analysis of Metabolites

**Figure 3:**
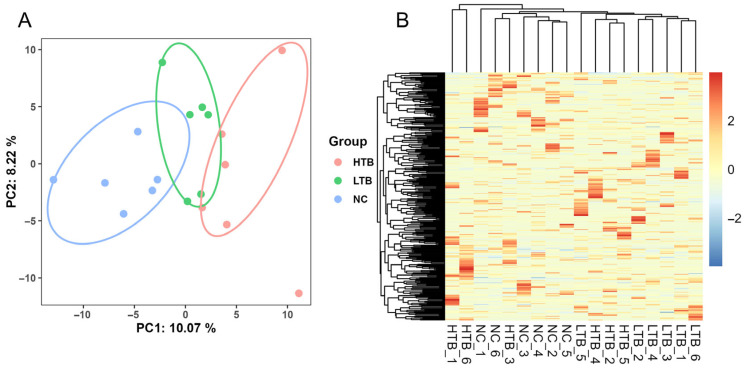
Multivariate analysis of the metabolome by PCA and hierarchical clustering. (**A**) PCA score plot of the metabolomic profiles. (**B**) Cluster analysis of the metabolite-based heatmap.

A total of 2174 metabolites were identified in this analysis. The dataset underwent preliminary screening based on the criterion that metabolites with missing values exceeding 80% in all groups were excluded. Ultimately, 321 metabolites were retained for subsequent analysis. To assess the metabolic impact of dietary TBs in grass carp muscle under freeze–thaw conditions, we employed principal component analysis (PCA). The analysis revealed that the first principal component (PC1) accounted for 10.07% of the total variance, while the second principal component (PC2) explained 8.22%. Samples within each group clustered closely, indicating low intragroup variability (Figure 3A). Following normalization of metabolite data, hierarchical cluster analysis was conducted across all samples. There was a clear grouping pattern of metabolites, indicating that the metabolites of grass carp muscle fed with TBs after freeze–thaw were significantly different from those of the control group (Figure 3B).

### 3.4. Analysis of OPLS-DA Model

**Figure 4:**
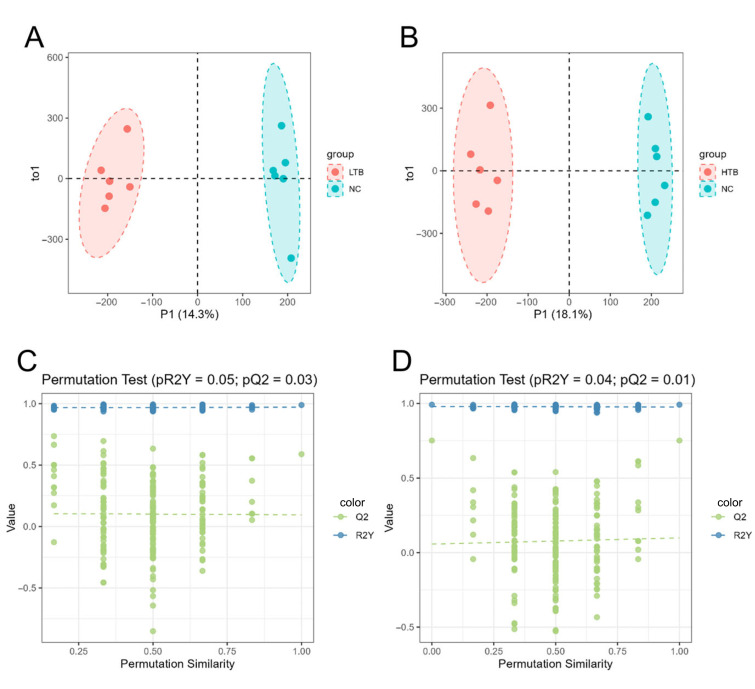
Orthogonal partial least squares discriminant analysis (OPLS-DA). (**A**) LTB vs. NC OPLS-DA score chart. (**B**) HTB vs. NC OPLS-DA score chart. (**C**) LTB vs. NC permutation test. (**D**) HTB vs. NC permutation test; the dotted line represents the actual values marked on the original model, and the horizontal axis indicates the degree of similarity between the statistics obtained after randomly replacing the sample labels and the original statistics.

To investigate the global metabolic alterations in fish flesh induced by dietary TBs during freeze–thaw processing, partial least squares discriminant analysis (PLS-DA) was applied. A clear separation among the NC, LTB and HTB groups was observed, indicating significant differences in metabolite profiles between the NC, LTB and HTB groups (Figure 4A,B). Furthermore, statistical validation of the PLS-DA models revealed significant *p*-values for both the explained variance (R2Y) and the predictive capacity (Q2). Specifically, in the LTB vs. NC, pR2Y = 0.05 and pQ2 = 0.03 (Figure 4C), while, in the HTB vs. NC, pR2Y = 0.04 and pQ2 = 0.01 (Figure 4D). These validation results confirm that the observed metabolic distinctions are statistically robust, demonstrating that dietary TBs significantly modulate the metabolomic profile of fish muscle following freeze–thaw cycles.

### 3.5. Analysis of Differences in Metabolites

**Figure 5:**
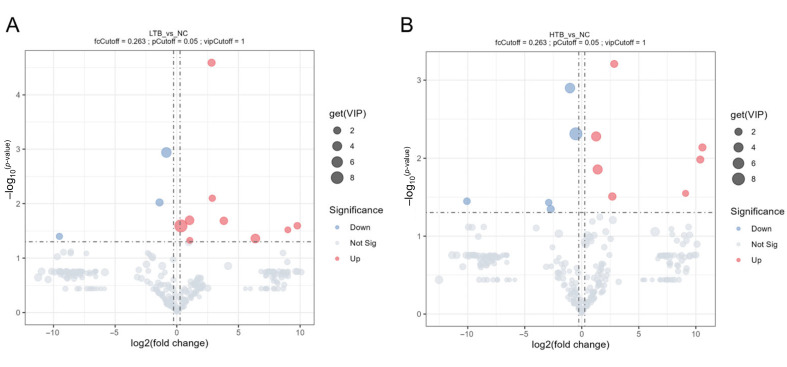
Differential volcano map. (**A**) The volcano diagram of the difference between LTB vs. NC. (**B**) The volcano diagram of the difference between HTB vs. NC; the gray parts are not significantly different, the red parts are significantly upregulated and the blue parts are significantly downregulated.

**Figure 6:**
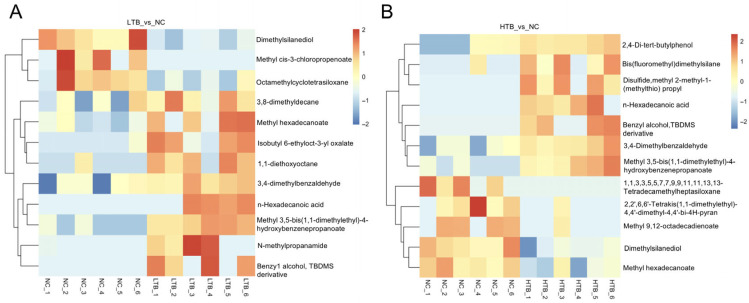
Heat map of differential metabolites. (**A**) Heat map of differential metabolites of LTB vs. NC. (**B**) Heat map of differential metabolites of HTB vs. NC; the colors corresponding to different metabolites reflect their abundance. The redder the color, the higher the abundance; the bluer the color, the lower the abundance.

Differential metabolites were screened using thresholds of a *p*-value < 0.05, |log2(Fold Change)| > 0.263, and VIP > 1. This analysis identified twelve differential metabolites in the LTB vs. NC (Figure 5A), with nine upregulated and three downregulated. In contrast, the HTB vs. NC (Figure 5B) yielded twelve metabolites, comprising seven upregulated and five downregulated. Notably, the main metabolites upregulated in the LTB group were fatty acids and esters, whereas those in the HTB group primarily comprised fatty acids and aromatic compounds (Figure 6A,B). These alterations in muscle metabolites indicate that dietary TBs may influence grass carp muscle quality, particularly in the context of freeze–thaw processing. The increase in aromatic compounds, potential flavor precursors, suggests a possible impact on the sensory profile of the fish (Table 2). Furthermore, the differential response between LTB and HTB groups implies that the effect of TBs on fish quality-related metabolism is concentration-dependent.

## 4. Discussion

This study aims to explore the potential application value of TBs in enhancing the quality of frozen foods by investigating the effects of dietary TBs supplementation on muscle texture, ice crystal morphology and the metabolic profile of grass carp during freeze–thaw cycles. We evaluated the effects of dietary TBs supplementation on the muscle quality of grass carp subjected to freeze–thaw cycles using texture analysis, histological and untargeted metabolomics approaches. The results demonstrate that the addition of TBs at a high concentration (0.06%TBs) effectively preserves the microstructural integrity of muscle tissue by significantly reducing ice crystal formation, thereby improving key textural properties of the fish muscle, including chewiness and cohesiveness. Furthermore, metabolomic analysis reveals that TBs exert a significant regulatory influence on the muscle metabolic profile. These findings indicate that the inclusion of TBs in feed improves muscle frost resistance through the regulation of endogenous metabolic processes, thereby establishing a foundation for future investigations into potential molecular mechanisms.

Texture characteristics represent a critical sensory attribute through which consumers perceive food via oral tactile sensations following visual and olfactory cues, significantly influencing overall food evaluation and consumer experience [34,35]. The cohesiveness of muscle reflects the internal binding strength of muscle, whereas chewiness refers to the energy required to masticate food into a swallowable state [36,37]. These textural attributes are closely associated with the structural organization of muscle fibers, the content and degree of cross-linking of intramuscular connective tissue (collagen) and the characteristics of myofibrillar proteins [38,39,40]. Recent studies indicate that dietary biotin can significantly enhance collagen deposition in grass carp muscle by transcriptionally activating genes involved in collagen synthesis and modulating the expression of genes related to collagen degradation, thereby improving muscle chewiness and hardness [41]. Similarly, dietary vitamin C has been shown to increase the hardness and springiness of grass carp muscle by promoting the synthesis of both collagen and elastin [42]. Our research findings indicate that supplementing grass carp feed with freeze–thaw cycles of TBs does not result in significant changes in muscle adhesiveness, hardness and springiness, while significantly increasing cohesiveness and chewiness. Taken together, these results suggest that TBs may have affected the collagen deposition or structural integrity of grass carp muscle through a similar pathway, thereby enhancing the cohesiveness and chewiness of the fish muscle.

The formation of ice crystals during freeze–thaw cycles is a critical factor influencing the quality of fish muscle. Ice crystal formation can disrupt tissue structure, promote lipid oxidation and rancidity and induce protein denaturation [43,44,45]. The formation of ice crystals during the freeze–thaw cycle in fish muscle is significantly influenced by intrinsic properties of the tissue, including water content and its distribution and collagen content [38,46]. Our research indicates that, after adding TBs to the feed, the ice crystals formed in freeze–thawed fish muscle are smaller and more uniformly distributed, which helps mitigate structural damage to the muscle tissue. In addition, antifreeze proteins in fish can synergize with collagen to effectively inhibit ice crystal growth during freezing, thereby offering enhanced and comprehensive protective effects [47,48]. Existing research indicates that antioxidants can enhance the expression of collagen-related genes and stimulate collagen synthesis in muscle tissue [41,42]. TBs, as a powerful antioxidant, may have functions similar to vitamin c, promoting the synthesis of collagen in muscles by activating the expression of collagen-related genes [49].

The quality alterations in fish muscle following freeze–thaw cycles have long been a focus of scientific research, as changes in metabolites directly influence the core attributes of fish muscle quality, such as freshness, flavor and tenderness [50]. During the freeze–thaw process, the formation and dissolution of ice crystals can disrupt the structural integrity of cell membranes, causing lipids such as phospholipids and triglycerides to be exposed to an oxidative environment [51]. This exposure renders lipid oxidation one of the primary factors contributing to the deterioration of aquatic product quality [52]. The oxidation process is typically characterized by an increase in reactive oxygen species (ROS), leading to the accumulation of malondialdehyde (MDA) and carbonyl compounds [53]. These oxidative byproducts are associated with undesirable changes in odor, color and the overall sensory attributes of aquatic products [51]. TBs are a polyphenolic high-molecular-weight compound exhibiting potent antioxidant properties. Existing studies have demonstrated that TBs can effectively reduce ROS levels and enhance antioxidant enzyme activity [54]. Metabolomic analysis was performed to investigate the alterations in metabolites following freeze–thaw cycles in grass carp fed with TB-supplemented diets. Our research indicates that the levels of esters and aromatic compounds in the LTB and HTB groups are significantly elevated. In summary, TBs may mitigate mechanical damage caused by ice crystals to cells during freeze–thaw cycles, preserve cell membrane integrity, and consequently reduce the oxidative degradation of ester compounds induced by oxidative stress throughout the freeze–thaw process. Furthermore, future research should prioritize conducting sensory evaluations by a professional panel. This approach will provide valuable and direct evidence for verifying the effects of TBs on fish muscle quality, ultimately enhancing the practical significance of the research outcomes for fish product development and quality control.

## 5. Conclusions

This study found that supplementing feed with TBs significantly improved the chewiness and cohesiveness of grass carp muscle. Furthermore, it effectively suppressed ice crystal growth during freeze–thaw processes, resulting in smaller and more uniformly distributed ice crystals. The addition of 0.06% TBs demonstrated a more pronounced effect compared to 0.02% TBs. Metabolomics analysis further indicated that the inclusion of TBs in the feed resulted in significant alterations in muscle metabolite profiles. These findings suggest that dietary TBs can mitigate freeze–thaw-induced damage by modulating the metabolic status of grass carp muscle, thereby offering novel insights and potential strategies for improving the preservation quality of frozen aquatic products.

## Figures and Tables

**Table 1:** Formulation and nutrient composition (%, dry matter).

Ingredients	Groups		
	NC	LTB	HTB
Wheat Flour	29.8	29.78	29.74
Soybean Meal	27	27	27
Rapeseed Meal	15	15	15
Peanut Hulls	12	12	12
Fish Meal	10	10	10
Fish Oil	2	2	2
Ca(H_2_PO_4_)_2_	1.5	1.5	1.5
Soybean Oil	1	1	1
Vitamins and Trace Elements ^a^	1	1	1
Choline Chloride	0.5	0.5	0.5
Antimicrobial Agents	0.2	0.2	0.2
Theabrownins	0	0.02	0.06

^a^ VA 10,800 IU, VD 34,000 IU, VE 40 IU, VK 33.4 mg, VB 11.6 mg, VB 212 mg, VB 66 mg, VB 120.05 mg, biotin 0.2 mg, folic acid 2 mg, niacin 50 mg, Ca 25 mg, Fe 80 mg, Cu 100 mg, Mn 50 mg, Zn 90 mg, Co 1 mg, Se 0.17 mg, I 0.15 mg. The antibacterial agent is a 1:1 mixture of calcium propionate and ammonium propionate.

**Table 2:** Detailed list of differentially expressed metabolites in different comparison groups.

Group	Up	Down
LTB_vs_NC	Isobutyl 6-ethyloct-3-yl oxalate; Methyl hexadecanoate; N-methylpropanamide; 3,8-dimethyldecane; Benzyl alcohol, TBDMS derivative; 1,1-diethoxyoctane; n-Hexadecanoic acid; 3,4-dimethylbenzaldehyde; Methyl 3,5-bis(1,1-dimethylethyl)-4-hydroxybenzenepropanoate	Methyl cis-3-chloropropenoate; Dimethylsilanediol; Octamethylcyclotetrasiloxane
HTB_vs_NC	Benzyl alcohol, TBDMS derivative; n-Hexadecanoic acid; 3,4-Dimethylbenzaldehyde; Methyl 3,5-bis(1,1-dimethylethyl)-4-hydroxybenzenepropanoate; Bis(fluoromethyl)dimethylsilane; Disulfide, methyl 2-methyl-1-(methylthio)propyl; 2,4-Di-tert-butylphenol;	2,2′,6,6′-Tetrakis(1,1-dimethylethyl)-4,4′-dimethyl-4,4′-bi-4H-pyran; Dimethylsilanediol; Methyl hexadecanoate; 1,1,3,3,5,5,7,7,9,9,11,11,13,13-Tetradecamethylheptasiloxane; Methyl 9,12-octadecadienoate

## Data Availability

The original contributions presented in the study are included in the article, further inquiries can be directed to the corresponding authors.
